# Editorial: Thyroid disorders in children below 3^rd^ year of life: Age-related specificity and challenges

**DOI:** 10.3389/fendo.2022.1029078

**Published:** 2022-10-05

**Authors:** Anna Małgorzata Kucharska, Małgorzata Gabriela Waśniewska

**Affiliations:** ^1^ Department of Pediatrics and Endocrinology, Medical University of Warsaw, Warsaw, Poland; ^2^ Department of Human Pathology in Adulthood and Childhood, University of Messina, Messina, Italy

**Keywords:** congenital hypothyroidism, neonatal hyperthyroidism, prematurity, thyroid disorders, young children

## Introduction

Thyroid disorders in children below 3 years old can lead to the deterioration of mental and physical development. This period is critical in the process of activity-dependent synaptogenesis and its plasticity (1) and thyroid hormones play a key role in this process. Furthermore, congenital hypothyroidism is the major cause of intellectual disability which could be completely preventable if diagnosed in time.

The breakthrough in this issue was the introduction of neonatal screening for congenital hypothyroidism established in many countries around the world. Considerable experience regarding diagnostics, treatment, and effects of this rare condition is currently available thanks to the collected data. Based on this experience, numerous articles concerning thyroid disorders have been published, thus improving the care of patients (2, 3). The recently updated guidelines from the European Reference Network on Rare Endocrine Conditions (ENDO-ERN) for congenital hypothyroidism (CH) determine the timing, dosage, and monitoring of the therapy (2). Nevertheless, some particular aspects of the disorder are still not entirely elucidated. A considerable challenge is the assessment of thyroid axes in preterm babies and neonates born by mothers with thyroid disease.

## Hypothyroidism before the 3^rd^ year of life

The most common cause of this condition is CH, which in the majority of cases is detected in the neonatal screening test. In children younger than 3 years old, when the brain development is still undergoing its critical phase, a deficiency as well as an excess of thyroid hormones could cause serious consequences. The proper treatment should be well balanced, avoiding undertreatment as well as thyrotoxicosis. The recommended initial dose of levothyroxine (LT4) is 10-15mcg/kg/d for each neonate with a decreased level of fT4 whereas, in children with fT4 within normal ranges, the initial dose could be 5-10mcg/kg/day (2, 3). These doses most likely avoid overtreatment. However, the optimal LT4 dosage is still under debate. In a Dutch study (4), it was found that both early over- and undertreatment may lead to permanent behavioral problems, the former to ADHD and the latter to autism spectrum disorders. It seems that a more precise individualization of dosage, according to the presence of the thyroid gland and initial thyroglobulin level, as well as the potential absorption and bioavailability of LT4 preparations should be considered. Concerning this issue in our Research Topic, Lipska et al. analyze the problems of treatment in 99 children diagnosed with primary CH and describe 5-years worth of experience considering under- and overtreatment. In addition, Esposito et al. analyze the effect of initial levothyroxine dose on neurodevelopmental and growth outcome in a group of children with CH in a prospective randomized trial and Stagi et al. describe the new possibilities of treatment with different LT4 formulations and varying factors influencing the process. Furthermore, Tuli et al. report the primary results of comparison between two liquid LT4 formulations in the treatment of CH.

Another issue is the prevalence of hypothyroidism in preterm newborns. A review of literature on this topic is presented by Kłosińska et al. As the complement to this review, two unrelated analyses based on large groups of preterm babies by Stawerska et al. and Mikołajczak et al. are published. In the latter paper, the authors report the unique data regarding thyroid volume and thyroid axis function in children born before 33 weeks of gestation. In spite of extensive data, there are still unsolved questions. The current recommendations define the indications for diagnostics and LT4 treatment in these children (2, 3, 5), but it is still necessary to update the knowledge in this area.

The thyroid dysfunction before 3 years of life is not always dependent on inborn defects or prematurity. Acquired autoimmune thyroiditis is also reported in very young children (6). Caprio et al. report a case of acquired overt hypothyroidism in a child in their second year of life dependent on iodine deficient hypoallergic diet. This condition should be considered in some children on special diet regardless of sufficient iodination of general population.

Silva et al. report the increased risk of Helicobacter pylori infection observed in children with CH and discuss the possible mechanisms influencing the relationships between these two conditions.

## Hyperthyroidism before the 3^rd^ year of life

Hyperthyroidism before the 3^rd^ year of life concerns the neonatal period in the majority of cases and is affiliated with maternal Graves’ disease (GD). It could originate from neonates’ mothers with active GD but also euthyroid with a history of GD. Severely affected children are at risk of craniosynostosis, cardiac insufficiency, and thyroid associated ophtalmopathy (TAO). The guidelines for the management of this condition define the anti-TSHR antibodies as the most important predictor of neonatal GD (7–9). In our Research Topic, Pyrżak et al. analyze a long follow-up of thyroid function and psychophysical development in children with neonatal hyperthyroidism while Dong et al. reporte the therapeutic perspectives in TAO in pediatric population.

## Conclusions

This Research Topic provides an important contribution to the discussion on thyroid disorders in very young children. Although hypo- and hyperthyroidism in children before the 3^rd^ year of life are both rare conditions, they can significantly affect the child’s future development and quality of life. Early diagnosis and adequate pharmacological treatment are effective means to resolve the alterations and avoid irreversible consequences of thyroid alterations in this particular age, as documented by the clinical studies presented in this Research Topic. Moreover, the collected papers demonstrate the need for further prospective studies with long-term follow-up concerning the effects on physical, intellectual, and behavioral development in children with early onset thyroid disorders.

## Author contributions

AK, MW conceptualized, designed, wrote, and approved the Editorial. All authors contributed to the article and approved the submitted version

## Acknowledgments

We thank every reviewers working on this Research Topic for their precious comments and help.

## Conflict of interest

The authors declare that the research was conducted in the absence of any commercial or financial relationships that could be construed as a potential conflict of interest.

## Publisher’s note

All claims expressed in this article are solely those of the authors and do not necessarily represent those of their affiliated organizations, or those of the publisher, the editors and the reviewers. Any product that may be evaluated in this article, or claim that may be made by its manufacturer, is not guaranteed or endorsed by the publisher.
